# The GummiArm Project: A Replicable and Variable-Stiffness Robot Arm for Experiments on Embodied AI

**DOI:** 10.3389/fnbot.2022.836772

**Published:** 2022-03-11

**Authors:** Martin F. Stoelen, Ricardo de Azambuja, Beatriz López Rodríguez, Fabio Bonsignorio, Angelo Cangelosi

**Affiliations:** ^1^Department of Computer Science, Electrical Engineering and Mathematical Sciences, Western Norway University of Applied Sciences, Bergen, Norway; ^2^Fieldwork Robotics Ltd., Cambridge, United Kingdom; ^3^Centre for Robotics and Neural Systems (CRNS), University of Plymouth, Plymouth, United Kingdom; ^4^MISTLab.ca, Polytechnique Montréal, Montreal, QC, Canada; ^5^Heron Robots, Genoa, Italy; ^6^Department of Computer Science, University of Manchester, Manchester, United Kingdom

**Keywords:** embodied intelligence, soft robotics, 3D printing, variable-stiffness actuators, replicable robotics research

## Abstract

Robots used in research on Embodied AI often need to physically explore the world, to fail in the process, and to develop from such experiences. Most research robots are unfortunately too stiff to safely absorb impacts, too expensive to repair if broken repeatedly, and are never operated without the red kill-switch prominently displayed. The GummiArm Project was intended to be an open-source “soft” robot arm with human-inspired tendon actuation, sufficient dexterity for simple manipulation tasks, and with an eye on enabling easy replication of robotics experiments. The arm offers variable-stiffness and damped actuation, which lowers the potential for damage, and which enables new research opportunities in Embodied AI. The arm structure is printable on hobby-grade 3D printers for ease of manufacture, exploits stretchable composite tendons for robustness to impacts, and has a repair-cycle of minutes when something does break. The material cost of the arm is less than $6000, while the full set of structural parts, the ones most likely to break, can be printed with less than $20 worth of plastic filament. All this promotes a concurrent approach to the design of “brain” and “body,” and can help increase productivity and reproducibility in Embodied AI research. In this work we describe the motivation for, and the development and application of, this 6 year project.

## 1. Introduction

### 1.1. The Need for Physical Robot Platforms for Embodied AI

The quest for hacking and recreating natural intelligence has seen plausible progress in the last decades, mainly driven by a major increase in computational power, and has often been developed using models or simulated environments. However, there is a claim, from different parts of the community pursuing this challenge, that progress will be slow unless real-world experiments are carried out, and that the role of the physical body, and its complex interaction with the environment, have been under-estimated. Perhaps even to the point that some of the latest progress is invalid and requires reviewing its assumptions. Some examples of this claim, can be found in concepts such as the symbol grounding problem (Harnad, 1990), and the replacement hypothesis (Shapiro, 2019), along with the work of Dennett (1994), Pfeifer and Bongard (2006), Bonsignorio (2007), Bonsignorio (2008), Wilson and Golonka (2013), and Eiben (2014).

There is a clear need for robust physical robotic platforms that enable the realization and reproduction of experiments in work on Embodied AI. For example in Embodied Cognition (Shapiro, 2019), where the body provides a resource for developing (cognitive) strategies to solve a task, and can help evoke or enlighten the formulation of hypotheses. Or in the field of Developmental Robotics (Cangelosi and Schlesinger, 2015), which requires platforms where computational models from psychology can be implemented and validated at human scale with tangible numbers and repeatable information (Cangelosi et al., 2015). Finally, in the field of Evolutionary Robotics, Eiben (2014), which requires a system that represents an approximation of the biological reality, able to exploit the richness of matter and capable of undergoing open-ended evolution in an open environment, to support the study of the co-evolution of minds and bodies.

As noted in Nygaard et al. (2019), designing such physical platforms is not an easy task. Such systems shall fulfill requirements like: operating under real-world conditions, being capable of interacting with the environment (including humans), capable of learning their own movements, allowing repeated and life-long experiments, among others.

**Figure 1:**
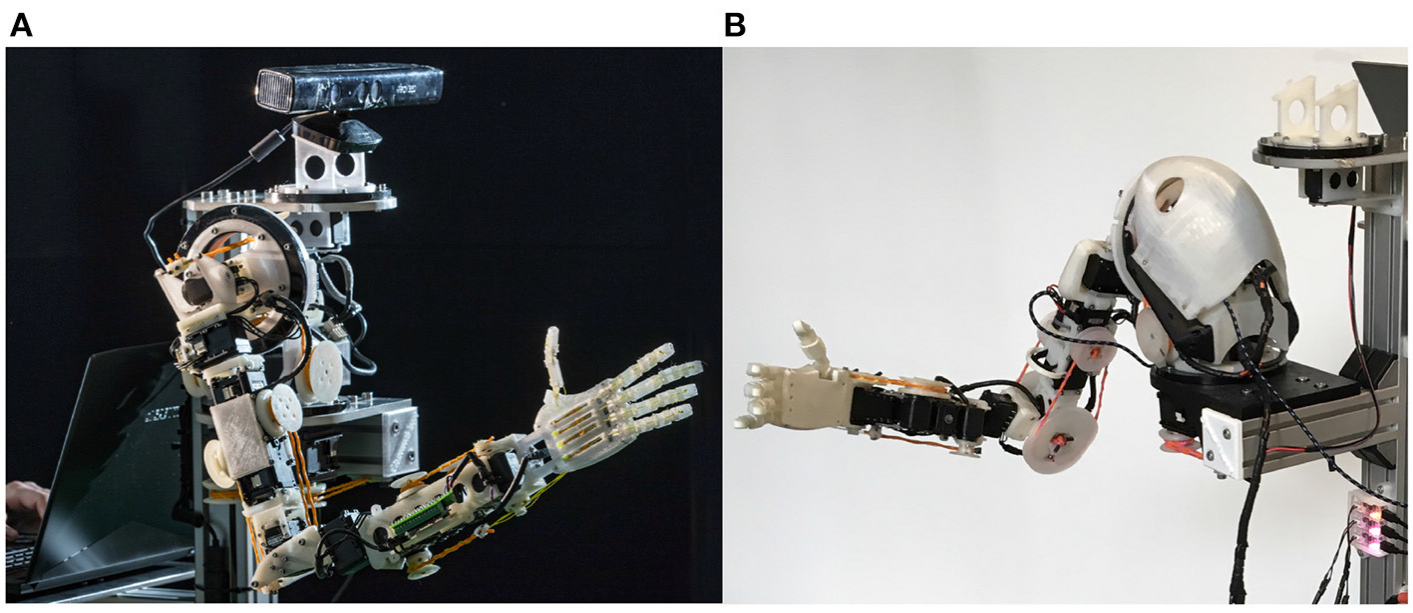
The original GummiArm 7+1 DOF robot arm with variable-stiffness is shown in **(A)**. The CE/RE version is shown in **(B)**. The project is open source and the arm is mostly 3D-printable, leading to easy replication. The arm dimensions are scaled to a 50th percentile female human as defined in NASA (1995). For all printable parts and code see: https://github.com/GummiArmCE.

In this article we present the GummiArm Project. As shown in Figure 1, the GummiArm is a 7+1 Degrees Of Freedom (DOF) robot arm, largely printable on hobby-grade 3D printers. The GummiArm project began with a 3D printer and some open source software and electronics, like many other maker projects on github.com or hackaday.io. However, the project gradually also acquired a scientific objective. That is, while there are many robot arms available for research groups in robotics, few offer the ability to concurrently develop the “brain” and the “body.” Even fewer enable researchers push the robot beyond its limits mechanically on a regular basis, or include soft joints. See Table 1 for a comparison of the main open-source robotic arms available to the research community.

**Table 1:** Comparison of a set of currently available replicable robot arms.

**Name**	**Performance**		**Joints**		**Replicability**		
	**Payload, kg**	**Reach, m**	**DOF**	**Soft (VSA)**	RP^*^	**Open HW**	**Cost, $**
GummiArm	0.5–1	0.7	7	5 (Yes)	FFF	CC BY-SA	~5730
Quigley et al. (2011)	2	>1	7	4 (No)	JET^**^	N/A	4135
Reachy^a^	0.5	0.65	7	0	FFF	CC BY-SA	<4000
Niryo One^b^	0.3	0.44	6	0	FFF	CC BY-NC-SA	1200
RBX1 - Remix^c^	0.25	?	6	0	FFF	CC BY-SA	800
Thor^d^	0.75	0.6	6	0	FFF	CC BY-SA	<450
Myorobotics Arm^e^	?	~1	4	4 (Yes)	SIN^***^	CC BY-4.0	?
VSA-CubeBot^f^	<0.5	0.4	4	4 (Yes)	STP	CC BY-4.0	?
H2Arm^g^	0.15	0.10	4	3 (Yes)	FFF	CC BY-4.0	225

*That is, robot arms that strive toward easily available and open-source hardware and software, and that can be built and maintained using commonly available low-cost rapid prototyping approaches. Costs relate to materials only, they do not include salaries for building and sourcing*.

* *Rapid prototyping approach*.

** *Jet cutting*.

*** *Laser sintering*.

a *https://www.pollen-robotics.com/reachy/*.

b *https://niryo.com/docs/niryo-one/*.

c *https://roboteurs.com*.

d *https://hackaday.io/project/12989-thor*.

e *https://roboy.org/partners/myrobotics-arm/*.

f *https://qbrobotics.com/it/prodotti/qbmove-kit-base/*.

g *https://ieee-dataport.org/open-access/h2arm-bsp-vs-pid-experiments*.

There is also an ongoing effort in making robotics research more easily replicable (Bonsignorio and Del Pobil, 2015). The GummiArm Project supported this effort, through open-source software and hardware, including a structure that is largely printable on standard Fused Filament Fabrication (FFF) 3D printers. For Embodied AI this means an experiment with the physical robot can more easily be replicated by others in the community, leading to a more scientific approach to benchmarking and comparing approaches, and potentially a higher academic impact.

### 1.2. Soft, Open-Source and Replicable Robot Arms

One of the many abilities that differentiate humans from animals and robots, is that of dexterous manipulation, “dexterity of robots is still far behind that of humans" (Siciliano and Khatib, 2016). Robot control has historically relied on models of the robot (rigid-body), the environment, and the interface between them. This has included two broad paradigms, a computationally-expensive global motion planner that solves the movement in open space, and a fine motion planner that deals directly with the interaction; reconciling the two, however, is an ongoing challenge, as global planners are unable to satisfy feedback requirements, and fine motion control is subject to local minima (Siciliano and Khatib, 2016). One approach to solve this issue is to take inspiration from natural agents, and outsource impacts and contacts outside the realm of traditional closed loop control, to passive elements such as soft materials in the structure, and elastic elements in the actuation (Pfeifer et al., 2007).

As stated in Rus and Tolley (2015), the use of soft materials in robotic agents enables new capabilities in safety, speed and agility. It also allows off-loading some of the algorithmic complexity from the “brain” to the “body” (Pfeifer et al., 2012). However, soft bodies can be hard to control with algorithms that assume the availability of exact models of the robot body, and new approaches to orchestrate the control of body-brain-environment are required.

The robotics community has produced a considerable plethora of soft robot arms, from non-articulated continuum arms such as those inspired by the octopus (Mazzolai et al., 2012), or the elephant trunk (Hannan and Walker, 2003), or more recent approaches with more load-bearing capacity (Zhao et al., 2019); to robots with stiff links, but elastic elements connecting the links and actuators, such as those using series elastic actuators (Pratt and Williamson, 1995). Examples of implementations of series elastic actuators can be found in the low-cost compliant actuator developed by Quigley et al. (2011), see details in Table 1, the light industrial robot Baxter (Rethink Robotics, Boston, USA), or the intrinsically safe (for its low inertia), BioRob arm (Lens et al., 2010). The spring elements provide compliance, but this cannot be varied in real-time, leading to difficulties when trying to control such robots on fast point-to-point movements.

As an alternative, Variable Stiffness Actuators (VSA), Vanderborght et al. (2013) and Grioli et al. (2015), provide better performance in terms of efficiency, robustness and adaptability. Then a trade-off can be made between guaranteeing human safety and achieving the best possible robotic performance (in terms of accuracy and speed) (Bicchi et al., 2005). A good example of a low-cost and flexible implementation of VSA can be found in the VSA-Cubebot (Catalano et al., 2011), available open source (Melo et al., 2014). See also Table 1 for its 4 DOF arm configuration. VSA-Cubebot's modular approach simplifies the design of a VSA arm, but it requires that the actuators are placed at the joints. Instead, actuator-external tendons can give the designer the flexibility to locate the heavy motors away from the joints, reducing the moment-arm, and thus the torques required. An example of the use of tendons in an anthropomorphic arm, with full VSA actuation, can be found on the DLR hand arm system (Grebenstein et al., 2011). This solution aims at human levels of scale and performance, with the accompanying high cost and complexity level. These problems: high production costs, non-modular, purpose oriented, and complex design, have also been faced by anthropomimetic robot efforts such as the Eccerobot (Wittmeier et al., 2013), inhibiting their reproduction (Richter et al., 2016).

The introduction of elastic elements in the actuation loop can make damping of end-point oscillations difficult. A solution to this problem has been proposed in Radulescu et al. (2012) with an electrically damped actuator. In Kashiri et al. (2016) physical damping was varied using a piezo-electric clutch and an advanced sliding control. Petit et al. (2014) approach this problem by using advanced torque control methods, however, this normally requires an accurate model of the robot.

If exact models of the body cannot easily be pre-defined by the designer, they can perhaps be learnt as part of the “development” of the robot. Early human development also includes exploration of own sensors and effectors, and their causal relationships with the rest of the body, and the external environment. This ranges from basic motor babbling to the generalization of previously learnt goal-directed movements (Cangelosi and Schlesinger, 2015). There are several open-source platforms exploring this general direction. For example Roboy (Pfeifer et al., 2013). This is a tendon-driven humanoid robot with passive compliance and force sensing directly in the muscle units. See the related Myorobotics Arm (Richter et al., 2016) in Table 1. This bio-mimetic approach offers the possibility to explore control strategies that respond to challenges similar to those encountered in the biological musculoskeletal system, using, for example, the spherical shoulder joint proposed by Richter et al. (2016). The iCub (Metta et al., 2008), likely the largest open humanoid robotics project, has been successfully used for research into embodied models of human learning of tasks, such as counting (Ruciński et al., 2012), or language acquisition and production (Hinaut et al., 2014; Garćıa et al., 2018). However, it lacks passive compliance, and is aimed at a much higher cost and complexity level.

Robot software is commonly made open source, in particular through the Robot Operating System (ROS). However, robot hardware designs are less frequently made publicly available with enough information for it to be easily reproduced in a research environment. The benefits of open source hardware includes the possibility of much more rigorous replication of robotics experiments. Making virtual experiments open source and replicable is simpler (Stoelen et al., 2015), but a physical platform is (and should be) more convincing. However, replicating a hardware design can be very challenging, for example if precision machining or highly skilled assembly is required. Luckily, 3D printing technology is now at a maturity and cost level that enables $400 printers to create parts suitable for many robot components. Examples of the use of 3D printing technology in an open source robotic platform can be found in the Poppy project (Lapeyre et al., 2014), or the more recent initiative for open robotics: https://open-dynamic-robot-initiative.github.io reporting working prototypes for dexterous object manipulation (Wüthrich et al., 2020) and legged locomotion (Grimminger et al., 2020).

The GummiArm project takes inspiration from a series of open source robot hardware implementations that have utilized 3D printing successfully. Among them, the 3D-printable hands from the OpenBionics initiative (Liarokapis et al., 2014) and the Yale OpenHand Project (Ma et al., 2013). Additionally, there's the Soft Robotics Toolkit (Holland et al., 2014), which documents a range of soft robotics components, and recent advances in 3D-printable hydraulics (MacCurdy et al., 2016). The GummiArm complements these efforts by providing a full-size, dexterous and bio-inspired robot arm that is soft, open source and printable.

## 2. Method

### 2.1. Key Features of the GummiArm

**Figure 2:**
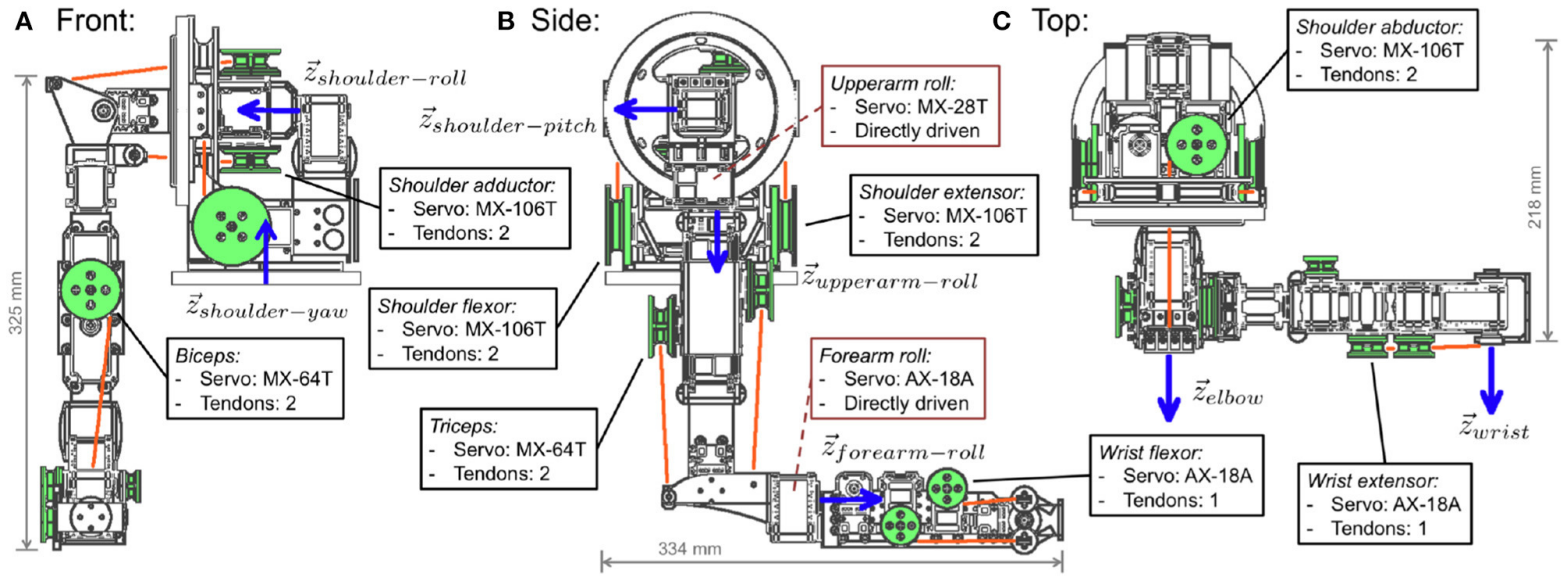
The structural layout of the GummiArm v1.0, seen from the front **(A)**, side **(B)**, and top **(C)**. The 7 joint axes ($\vec{z}$) are indicated with thick blue arrows. The tendons are shown as orange lines, while the servo pulleys are highlighted in green (8 shown, corresponding to the 4 out of 5 antagonist joints). Resting pose shown (zero degrees on all joints). Note that the structure and servos corresponding to the *shoulder yaw* joint are not shown for clarity.

The structure of the GummiArm consists of specially designed 3D printed plastic parts connected to Dynamixel digital servos of Robotis Inc (Irvine, CA, USA), as shown in Figure 2. This approach was partly inspired by the Robotis Bioloid robots and the Poppy Project (Lapeyre et al., 2014), using the same principles of modular, open source, 3D printed, and the same servomotor family, but with the addition of variable stiffness. That is, to introduce passive compliance in the joints so that damage to the servomotors would be reduced while experiencing partially unknown physical interaction, while also reducing the need to repair the non-flexible parts of the structure unnecessarily. High stiffness can still be used to perform tasks where a higher accuracy and movement stability is required.

Actuation of the joints in the GummiArm is mostly achieved by means of pairs of composite tendons in an agonist-antagonist configuration. These tendons are driven by a pulley on each servo-motor, and the servos are configured to work in agonist-antagonist pairs for most of the joints of the arm, providing the ability to vary the stiffness of these joints. The elasticity of the tendons is key to the ability to vary the stiffness. For agonist-antagonist VSAs, a spring with a quadratic increase in stiffness with elongation provides independent control of stiffness and position without sensory feedback, as shown in Ham et al. (2009).

**Figure 3:**
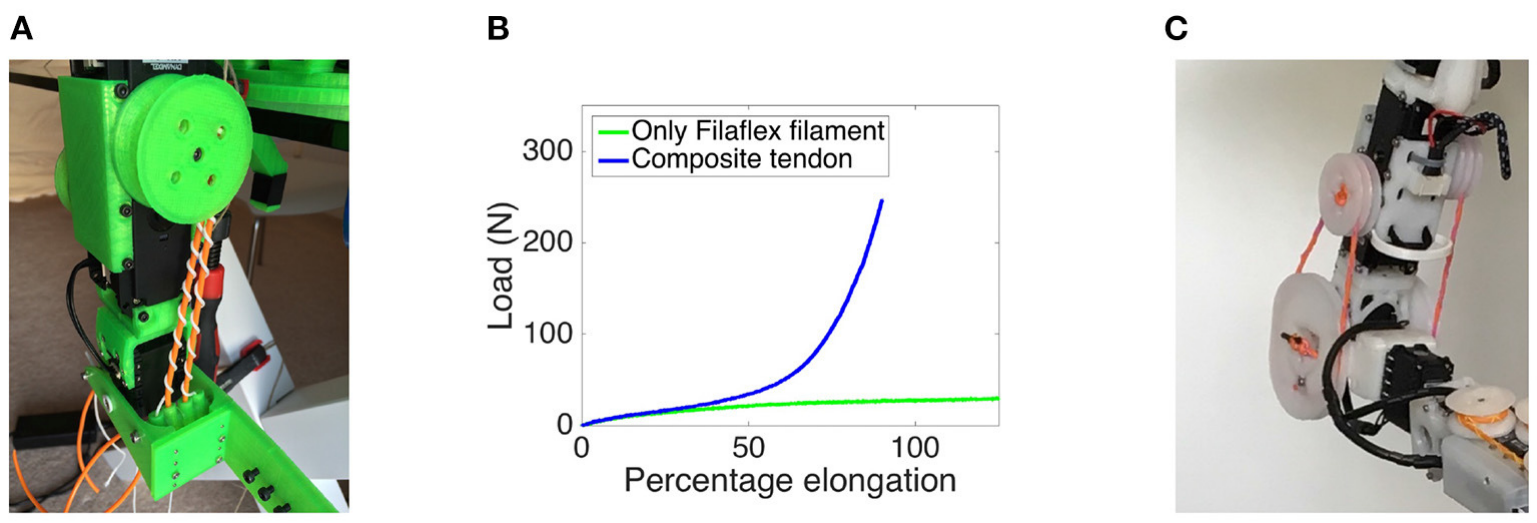
The composite rubber/nylon tendons on the *elbow* joint of the GummiArm v1.0 are shown in **(A)**, highlighting the *biceps* servo and tendons. Note the uni-directional agonist-antagonist setup, with the tendon set attached to one side of one actuated pulley. The tendons are composed of a soft 2.85mm Filaflex filament (Recreus, La Torreta, Spain) and a stiff 1.5 mm nylon thread twinned around it. A pitch of 0.1 revolutions per mm was used for the tensile test performed here. The results are shown in **(B)**, where the non-linear spring response of the tendons can be clearly seen. The testing was performed on an Instron (Wycombe, United Kingdom) 5582 frame with a static 100kN load cell (Instron UK195). Load (y-axis) vs elongation (x-axis) of tendons shown, comparing the Filaflex filament by itself with the composite tendon design. In **(C)** the *elbow* joint for the GummiArmCE/RE is shown for comparison, showing the bi-directional agonist-antagonist setup, where each servo can influence the movement of the joint in both directions, and the joint stiffness.

The tendons for the GummiArm have a flexible core of Filaflex 2.85 mm filament from Recreus (La Torreta, Spain). Around this core a nylon thread is spun, changing the original behavior of the material, allowing the stiffness of the tendon to increase with elongation, see Figure 3. For the tensile testing a pitch of 0.1 per mm was used. As can be seen in Figure 3B this composite tendon design has an increase in stiffness with elongation, as the nylon line gradually straightens out. The linear relationship up to 15% elongation can be largely avoided by pre-tensioning the tendons, which also reduces the risk of the tendons being displaced from the pulleys. The remaining force-length relationship has a fit of *R*^2^ = 0.94 with an exponential law of *F* = 5.718*e*^0.039*x*^. The viscoelastic nature of the rubbery core also means the tendons provide damping for the joint, which a metallic spring would not.

The Dynamixel servos have an engineered plastic or metal structure with solid mounting points, which means they can be used as load-bearing elements in the design. They can also be daisy-chained, which reduces the wiring to one set of cables between each successive servo in the arm. In the original GummiArm (here denoted GummiArm v1.0), the servos are joined by Polylactic Acid (PLA) plastic parts that can be printed on low-cost 3D printers.

A similar, but more complex, open source solution can be found in the Myorobotics Arm, https://roboy.org/partners/myrobotics-arm/, which mimics the complexity of the human arm (Richter et al., 2016). The solution has musculoskeletal properties, i.e., motors connect to other rigid parts only via tendons, which are in turn connected to springs in a triangular manner, to achieve a non linear force effect (enabling variable stiffness). Musculoskeletal robots provide built-in compliance and human-like weight distribution (mass of arm shifted toward the torso), and dynamics, which contributes to having safer interactions with humans and the environment. These properties are also found in the GummiArm, but the more traditional 1 DOF joints with built-in encoders simplify the control of the GummiArm considerably. The composite tendons also allow standard position-controlled rotational servomotors to be used.

As shown in Table 1, the overall cost of the parts for the GummiArm is less than $6000, 90% of which is incurred by the servomotors. The 0.5 kg of plastic needed to print a complete set of structural parts only costs about $20 (if PLA), a small fraction of the total cost. The passive compliance of the tendons makes the arm robust to high-bandwidth interactions with the environment, and a broken plastic part can be 3D printed (and potentially improved upon) quickly, and very cheaply. This also means the expensive servo motors are much less likely to be damaged. The GummiArm's total material cost is low enough to fit in the typical funding available to most PhD students, while being robust enough to be (regularly) run through thousands of trials interacting with real objects, and simple enough to be quickly fixed by students when it does break.

**Figure 4:**
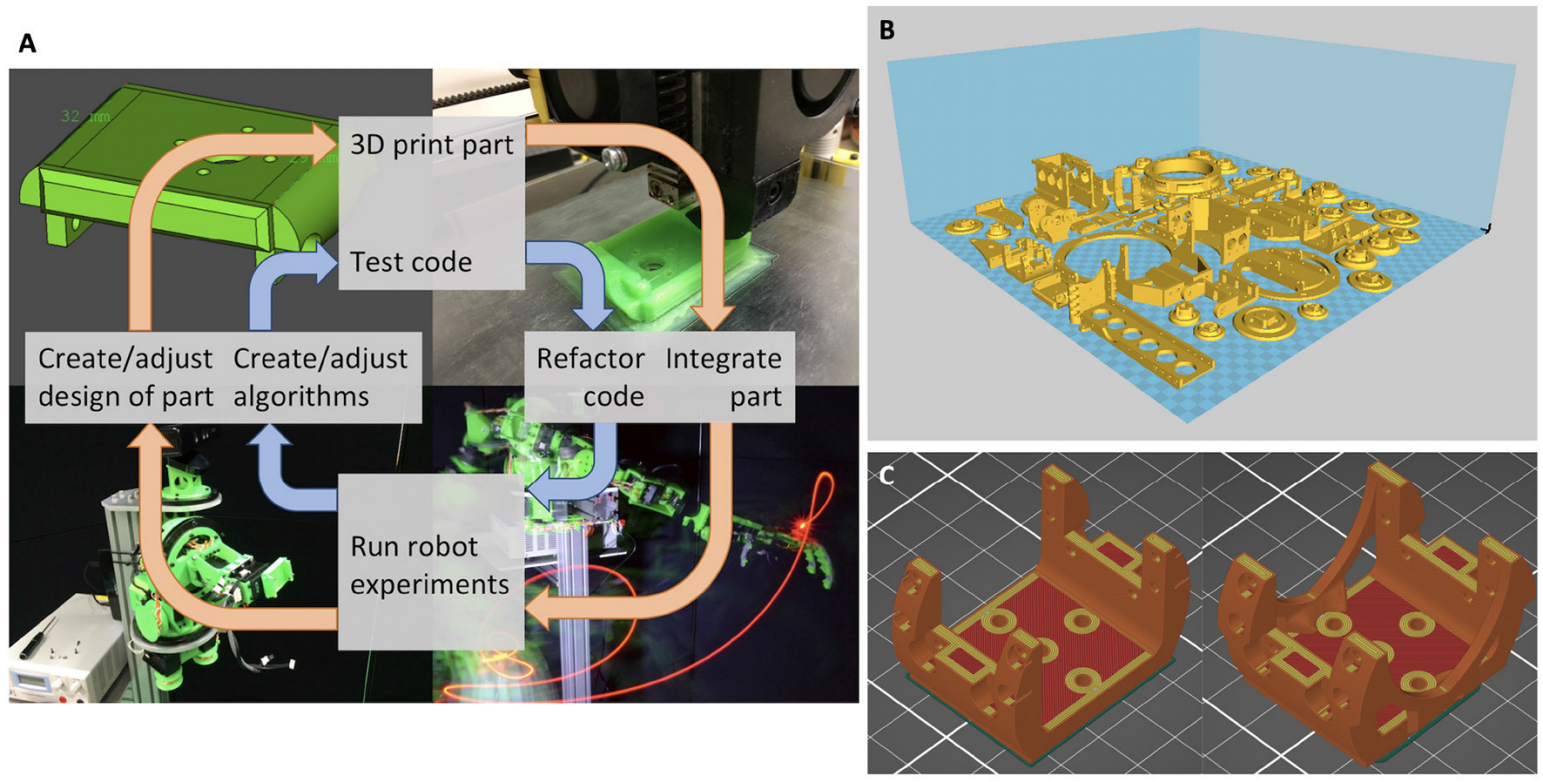
In **(A)**: 3D-printable hardware enables fast iterative design of both “brain” and “body.” The diagram shows the concurrent design loop of software (blue inner arrows) and hardware (orange outer arrows) of the GummiArm project. One complete cycle can take as little as just a few minutes. In **(B)**: The printable parts of the GummiArm v1.0, on a hypothetical 500 mm by 500 mm printing surface. Most parts are printed with 20% infill, which means a total of 523 g of PLA plastic is required. This is equivalent to 175 m of a 1.75 mm filament, with a total cost of about $20. On an original Prusa i3 MK3 with a print bed of 250 x 210 mm the following printing times are estimated: 16 h for parts required for the lower shoulder, 34 h and 23 min for parts required for the upper shoulder, 13 h for parts required for the lower arm, 13 h and 40 min for parts required for the upper arm, which makes a total of 77 h and 10 min. This is using a PrusaSlicer 2.3.0 based on Slic3r, with a 0.4 mm nozzle, in simple mode, using the default print settings “0.15 mm QUALITY,” modified to a 20% infill. In **(C)**: Change to the shoulder encoder holder for the GummiArmCE. On the left is the original part designed for PLA, while on the right is the Nylon version. The volume increased around 25% from PLA (12,358 mm^3^) to Nylon (15,437 mm^3^) and the weight went from 15.32 to 18.53 g, respectively. Sliced using PrusaSlicer 2.3.0 with 0.15 mm layer height, first layer 0.2 mm, minimum 5 vertical shells, minimum 7 top and 5 bottom solid layers, and 100% infill due to the high loading of the part.

The printable structure also opens up exciting possibilities for developing the hardware and software in parallel. This advantage is exploited by natural evolution, and seems especially important for softer robots, where software and hardware become highly interdependent (Bezzo et al., 2015). We believe such an approach is beneficial, both for exploring designs inspired by nature (and therefore, evolution), and for enabling robust robotic solutions to real-world problems. See Figure 4A for a representation of the concurrent design loop followed for the GummiArm project. A concurrent change to the 3D-printed body and the Python-based code can take as little as just a few minutes. This has led to a highly iterative form of design, where live experiments are part of each design cycle.

### 2.2. GummiArm v1.0

The original GummiArm had 7 actuated joints for performing arm movements, as seen in Figure 2. It also has a loosely anthropomorphic kinematic structure and actuator-tendon system, by using uni-directional agonist-antagonist VSAs. That is, with one tendon from each servo pulley only, meaning each servo can pull the joint in one direction only, similar to the muscles in the human arm. The joint angles were measured using standard Dynamixel AX-12 servos situated on the joint axis, but with the gearing removed (hence enabling the encoder to move freely with the joint). See Figure 4B for the full set of 3D printable PLA parts for GummiArm v1.0. PLA is cheap and easy to 3D print (it doesn't require a heated printing bed), and some researchers suggest printing with it generates less toxic fumes than other common plastics (e.g., Acrylonitrile Butadiene Styrene-ABS Wojtyła et al., 2017; Zhang et al., 2019). Moreover, 3D printed parts can be made relatively light and strong due to the non-solid internal infill structure. It has been shown that the influence of the different printing patterns (line, rectilinear and honeycomb) account for less than 5% in the maximum tensile strength but that the elastic modulus can be varied by almost 40% (Fernandez-Vicente et al., 2016). Using PLA, the total mass of the 7 DOF arm below the shoulder was 1.1 kg, and the total mass of the arm was 3 kg.

The *shoulder yaw, shoulder roll, shoulder pitch, elbow* and *wrist pitch* joints have antagonistic actuators, while the *upperarm roll* and *forearm roll* joints are directly driven by servos. All are controlled over two USB2Dynamixel interfaces from Robotis. As the servos represent more than 64% of the overall mass of the arm below the shoulder, the tendons help keep this mass as far as possible toward the shoulder, thereby reducing the torque required in the elbow and shoulder joints. The length of the upper arm from the top of the shoulder to the back part of the elbow is around 300 mm, equivalent to a 50th percentile female human. See measure 751 “shoulder-elbow length" in the NASA Man-Systems Integration Standards (NASA, 1995). With a stretched out hand, the total “forearm hand length" (measure 381 in NASA, 1995) is similar to a 50th percentile female human as well (i.e., around 400 mm).

**Figure 5:**
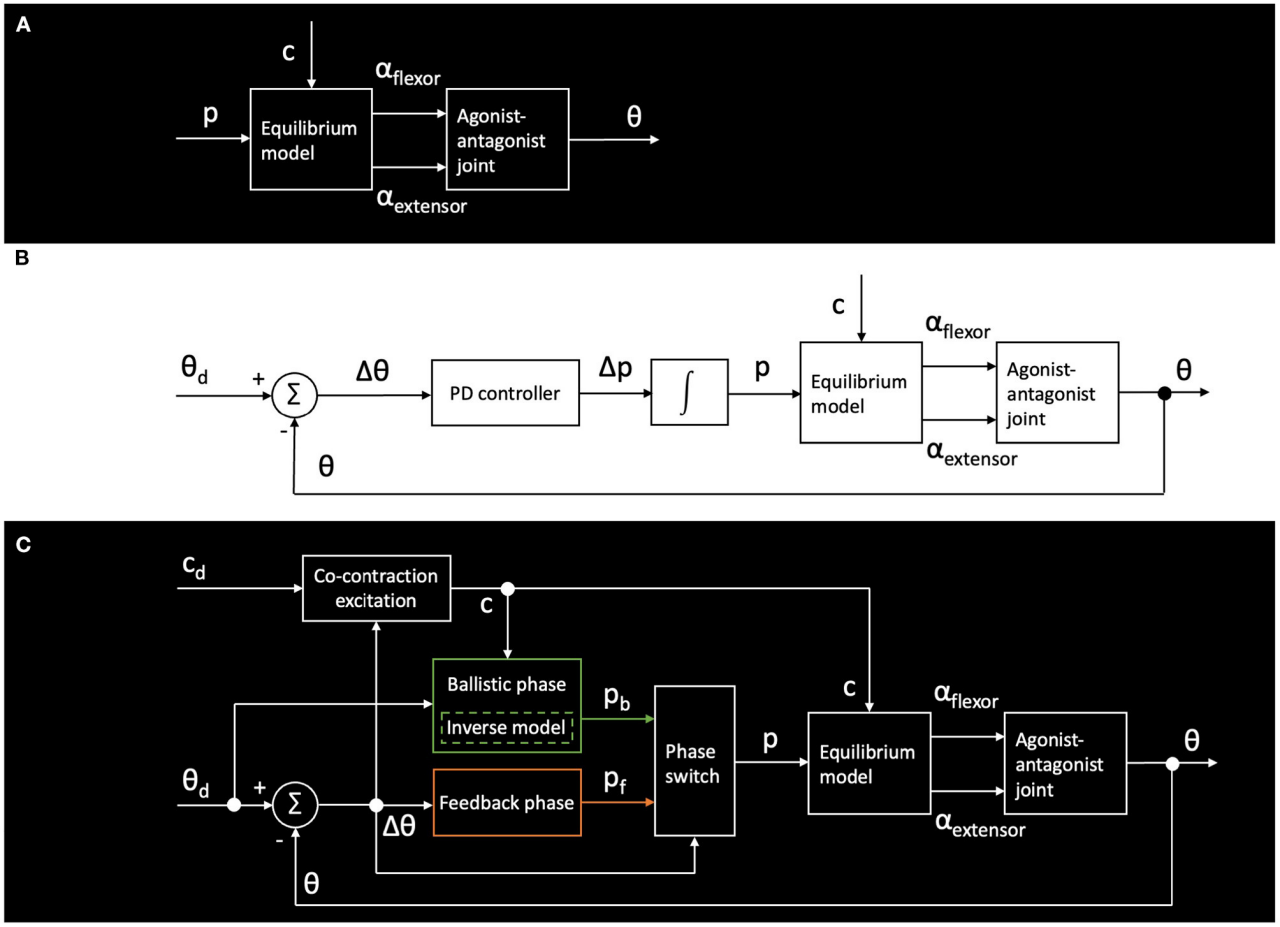
Example control architectures (for individual joints) used for the GummiArm experiments described here. **(A)** shows open-loop control using the equilibrium point *p* of and co-contraction level *c* of the joint. **(B)** shows a standard closed-loop approach with Proportional-Derivative (PD) control. **(C)** shows a bio-inspired dual-phase control for fast ballistic movements, as described in Stoelen et al. (2016).


The GummiArm is an interesting platform from a control perspective, given its variable-stiffness joints, but also the bio-inspired arrangement of the soft composite tendons. See Figure 5 for an overview of the joint-level control approaches used for the experiments performed in this paper. The two servo actuator angles for the flexor and extensor in the agonist-antagonist joints are defined as α_*flexor*_ and α_*extensor*_. See Eq. 1 for how these angles are calculated in the low-level GummiArm control. This relationship is hereby denoted as the “equilibrium model,” drawing inspiration from the human motor control literature (for example Bizzi et al., 1984), but without a direct analogy.

(1) $$\begin{array}{r} \begin{array}{c} \alpha_{\text{flexor}}=p\frac{\gamma }{4}-c\frac{\pi }{2},\\ \alpha_{\text{extensor}}=p\frac{\gamma }{4}+c\frac{\pi }{2}. \end{array} \end{array}$$

The variable *p* represents an equilibrium point for the joint, i.e., how far the joint has been moved in one direction or the other, through a concerted movement of the two servo actuators. The variable *c* represents the co-contraction of the joint, i.e., how much the two servo actuators have opposed each other, thus affecting the joint stiffness. It can be seen that the α_*flexor*_ and α_*extensor*_ for a joint is assumed to scale linearly with equilibrium point *p* and co-contraction *c*, for simplicity. The equilibrium point *p* ranges from −1 to 1, and is typically assumed to influence half the actuator range γ. This range depends on the joint, but is typically assumed to be several full rotations of the servo, exploiting the multi-turn feature of the Dynamixel servos used in the GummiArm. The co-contraction *c* ranges from 0 to 1 (corresponding to 0% to 100% co-contraction).

In Figure 5A the most basic scheme for open-loop control using the equilibrium point *p* of and co-contraction level *c* of the joint is shown. That is, these two variables completely describe a given set of actuator angles, which together with the loading on the joint determines the actual joint angle. This is useful for maintaining the arm passively compliant during physical interaction, and is among other exploited in the experiment on teleoperated control of door opening in Section 3.3. In Figure 5B a standard closed-loop approach with Proportional-Derivative (PD) control is shown. That is, where the equilibrium point *p* is shifted by the controller based on the joint-angle read from the encoder θ, and the desired angle θ_*d*_. This is the standard control approach used when following pre-planned trajectories. **C** shows a bio-inspired dual-phase control for fast ballistic movements, as described in Stoelen et al. (2016). Both are compared for step inputs in Section 3.4.

The project was hosted on GitHub at: https://github.com/mstoelen/GummiArm. This included Robot Operating System (ROS) compatible code for running the arm, mainly written in Python and C++, but also all the printable CAD models, and a wiki for documenting how to build, maintain and operate the arm. Note that the repository is now archived on GitHub, meaning it is read-only, and was superceded by the GummiArmRE/CE described in the next section. An open-source and antropomorphic hand design, with one actuator in the lower arm closing all fingers, was designed and integrated on the arm, inspired by the soft-robotic-hand design available here: https://github.com/MarcAntoineCRUE/Soft-robotic-hand.

### 2.3. GummiArmCE/RE

The GummiArmCE/RE can be seen in Figure 1B. In 2017, a new organization was created on GitHub to better host the growing community for the GummiArm. This is available at: https://github.com/GummiArmCE. The naming convention used from this point onwards was to distinguish between a Community Edition (CE) and a Research Edition (RE). Both were kept open source, but the RE was made available as a built and tested product with some modifications over the CE version. Here we will largely describe the two as the same, as the RE version is no longer offered commercially. The different clones of the arms were organized as branches of the relevant repositories for robot hardware and software parameters.

One of the main issues found with GummiArm v1.0 was the low torque available in the elbow joint, which limited the payload and caused overheating issues in the servos actuating the joint. This joint was therefore the first joint to be made bi-directional, i.e., where each servo pulley had tendons moving the joint in both directions. See Figure 1 for a visual comparison of the two versions of the elbow joint. This is a well-known configuration for agonist-antagonist joints, see Petit et al. (2010) for more details. It enables the robot to utilize the complete torque available from both servos to move the joint, when in a low-stiffness configuration.

To reduce the voltage drop across the many servos utilized in a daisy-chain fashion on GummiArm v1.0, and to ensure better stability in the power supply to the servos, the CE/RE version had a wiring loom that powered all servos in parallel. The drawback was an increase in the complexity of replicating the arm, with some electronics skills like soldering required.

In addition to redesigning and reinforcing parts that were seen as problematic on the GummiArm v1.0, some selected parts were also optimized for nylon, rather than PLA. While PLA is an easy-to-use, safer and stiff plastic for FFF, it has a relatively low fracture toughness. That is, it tends to fail catastrophically when overloaded, and especially across the layers in the 3D print. Nylon has a higher cost, and higher difficulty in 3D printing, but, when compared to PLA, or ABS, Nylon exhibits higher impact strength (Wickramasinghe et al., 2020), and is therefore less prone to fracturing. In the end a high-strength and low-shrinkage nylon-based filament of type Alloy 910 by Taulman (Linton, USA) was preferred. Note that recent advances in filaments, such as carbon reinforced nylon, means much better results can be achieved now, in terms of combining stiffness, strength and toughness. Given the lower stiffness of nylon, some parts had to be made more voluminous, but the overall mass and size of the arm remained similar to the GummiArm v1.0. As an example of the changes in volume and mass when the design migrated from PLA to Nylon, the shoulder encoder holder (Figure 4C) went from 12,358 mm^3^ and 15.32 g for PLA to 1,5437 mm^3^ and 18.53 g for Nylon at 100% infill and 0.15 mm layer height.

Finally, the arm was made more modular both in hardware and software, to help support the growing community and variety of uses for the arm. This included a split of the arm into *base* and *EE* (End Effector) both in software, and using quick-swap connectors for mechanical, electrical and communications. The *EE* was, somewhat confusingly, used to denote the complete lower arm and gripper, i.e., the quick-swap connection was made after the elbow joint. Another quick-swap mechanical connector was later added at the wrist enabling the use of commercially available grippers on the GummiArmCE/RE (e.g., The Universal Gummi Gripper: https://www.robotriks.co.uk/universal-gummi-gripper).

## 3. Results

The GummiArm has been used in a range of experiments, the complexity of which have increased with the maturity of the arm. We here give an overview of some of these experiments, focusing on the salient features of the arm such as co-contraction, ballistic movements, impact absorption, and physical interaction with the world during teleoperation. We hope to show how the experiments have helped drive the concurrent design process forward, and helped explore the full design space of hardware and software. An overview of experiments where the GummiArm has been replicated and used in the literature is also provided.

### 3.1. Demonstrating the Effect of Co-contraction

**Figure 6:**
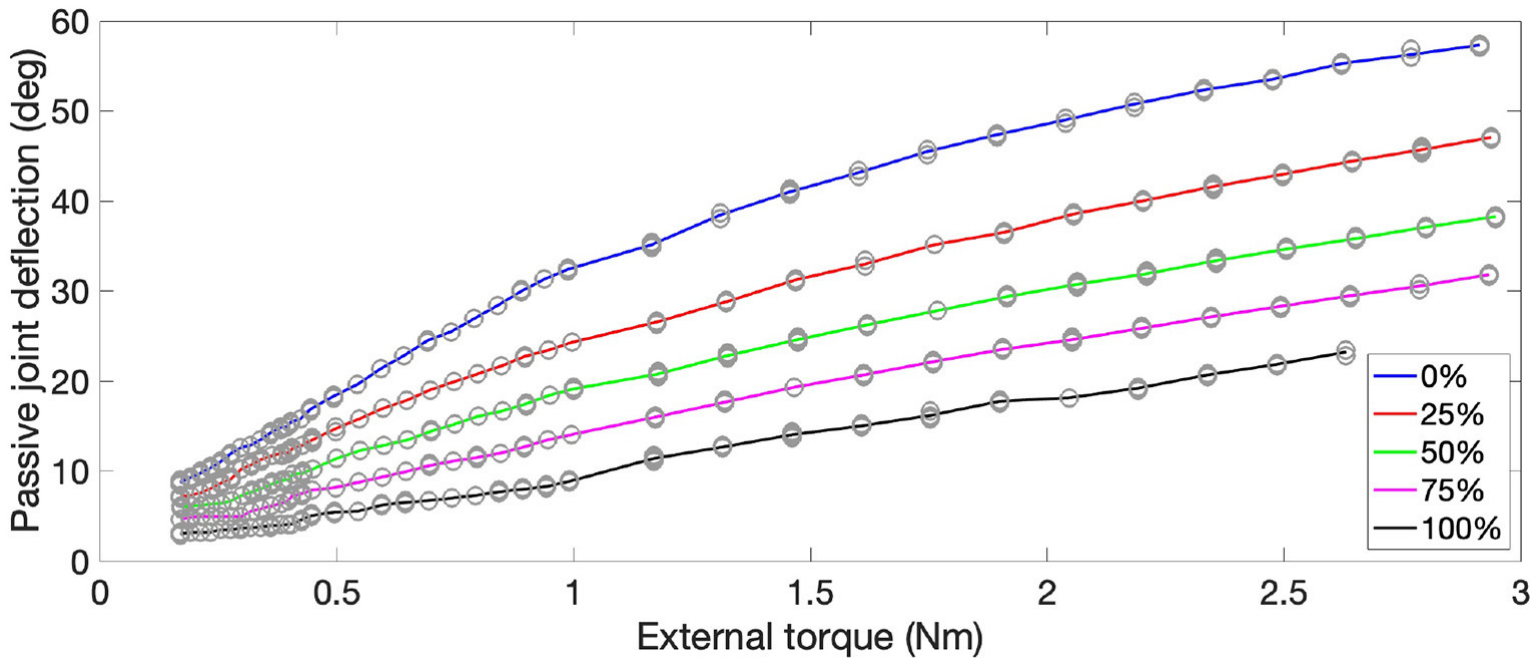
The composite rubber/nylon tendons provide an increase in stiffness with increase in co-contraction on the agonist-antagonist actuators of the GummiArm v1.0. The plot shows passive joint deflection (y-axis) of the *elbow* joint from applying an external torque (x-axis) under quasi-static conditions, for different values of commanded co-contraction. Deflection corresponding to elbow extension. The mean over 3 repetitions shown as solid lines, while open circles indicate data points.

This experiment show the ability to vary the stiffness of the bio-inspired joints by controlling the co-contraction of the antagonist actuators. A quasi-static loading setup was created for the *elbow* joint of the GummiArm v1.0. The upper arm was locked in place, while the lower arm was replaced with a rigid beam with multiple attachment points for weights, from 70 to 200 mm from the joint axis, and at 10 mm intervals. The actuator was commanded to a passive horizontal pose. Three different weights (0.1, 0.5, and 1.5 kg) were attached at different distances from the joint axis to generate a set of torques. The passive deflection of the joint was then recorded, and the process was repeated 3 times for the 3 weights and the 14 distances. The same procedure was repeated for 5 different co-contraction levels, from 0 to 100%. See Figure 6 for the results. The maximum torque feasible for the *elbow* joint with the Dynamixel MX-64T servo is close to 3 Nm, reducing somewhat with the highest co-contraction setting due to the counteraction of the opposing actuator. It can be seen that the amount of deflection for a given external torque can be changed considerably by the co-contraction, and the deflections possible are also quite high for a VSA (Grioli et al., 2015). Such “softness” is an interesting property for robots that explore autonomously the physical world. For example for the “motor babbling” experiments in developmental robotics (Cangelosi and Schlesinger, 2015).

### 3.2. Exploring the Relationship of Co-contraction, Damping and Overshoot

**Figure 7:**
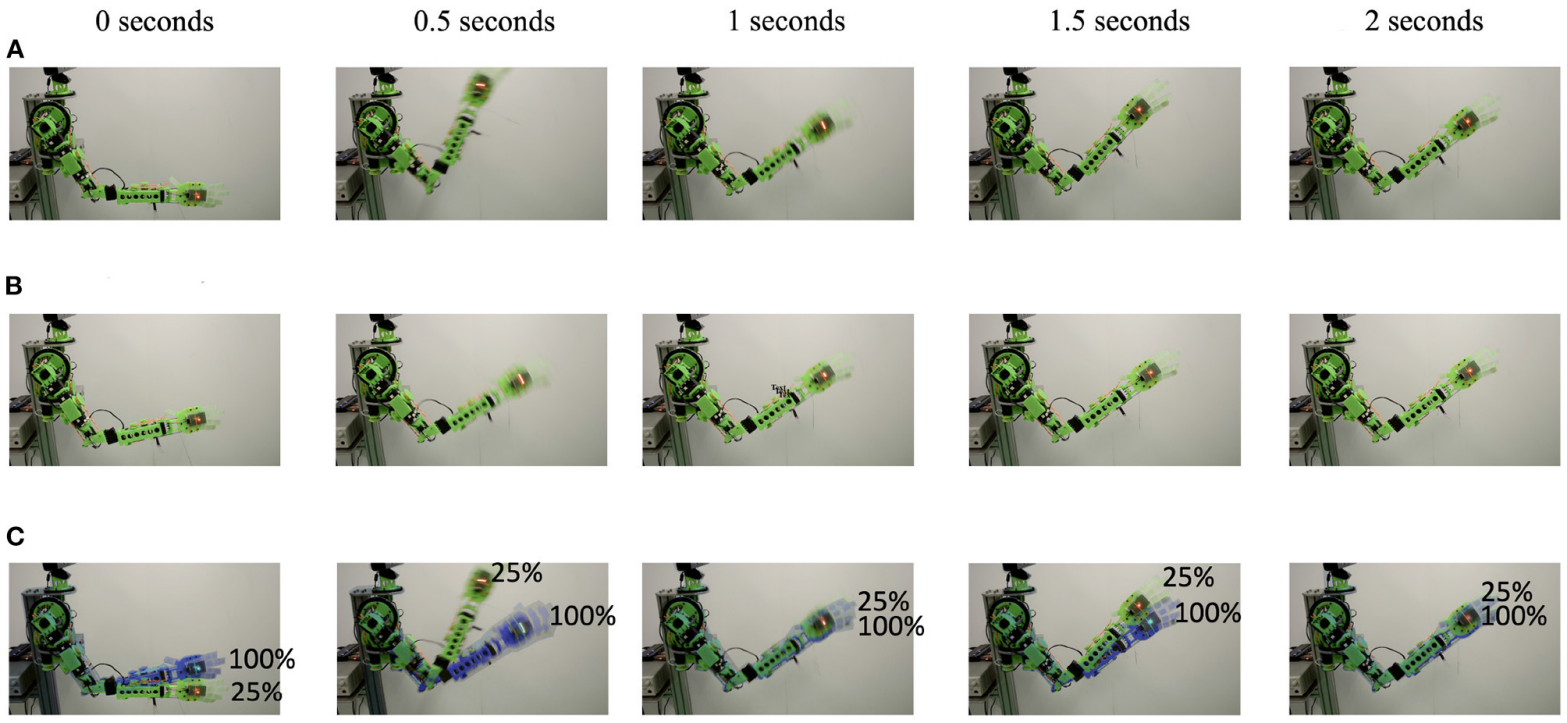
**(A)** Elastic energy is stored, but the robot arm response is quickly damped, even with low levels of actuator co-contraction. Thus the risk of damage and injury from such a release is reduced. Image series of a 1 kg mass release for 25% co-contraction. **(B)** Co-contraction can be used to limit the robot arm response to an elastic release. That is, the antagonist tendon can be kept taut, but not loaded, to react passively to the release of a force loading the agonist tendon. Image series of passive response to 1 kg mass release for 100% co-contraction. **(C)** Shows the overlapping image series from **(A,B)**.

Stored elastic energy can be harmful if released in an uncontrolled fashion. Damping can help reduce the oscillations caused by such a release, and co-contraction can help limit the overshoot by maintaining the opposing tendon taut. In a second experiment, the GummiArm v1.0 was loaded with a 1 kg mass at the wrist while extended forward. See Figures 7A,C. The arm was commanded to a given joint angle, then set in passive mode (i.e., maintaining the actuator servos at a fixed angle, but not closing the loop over the joint encoder), and loaded. The mass was then released, and the response recorded.

**Figure 8:**
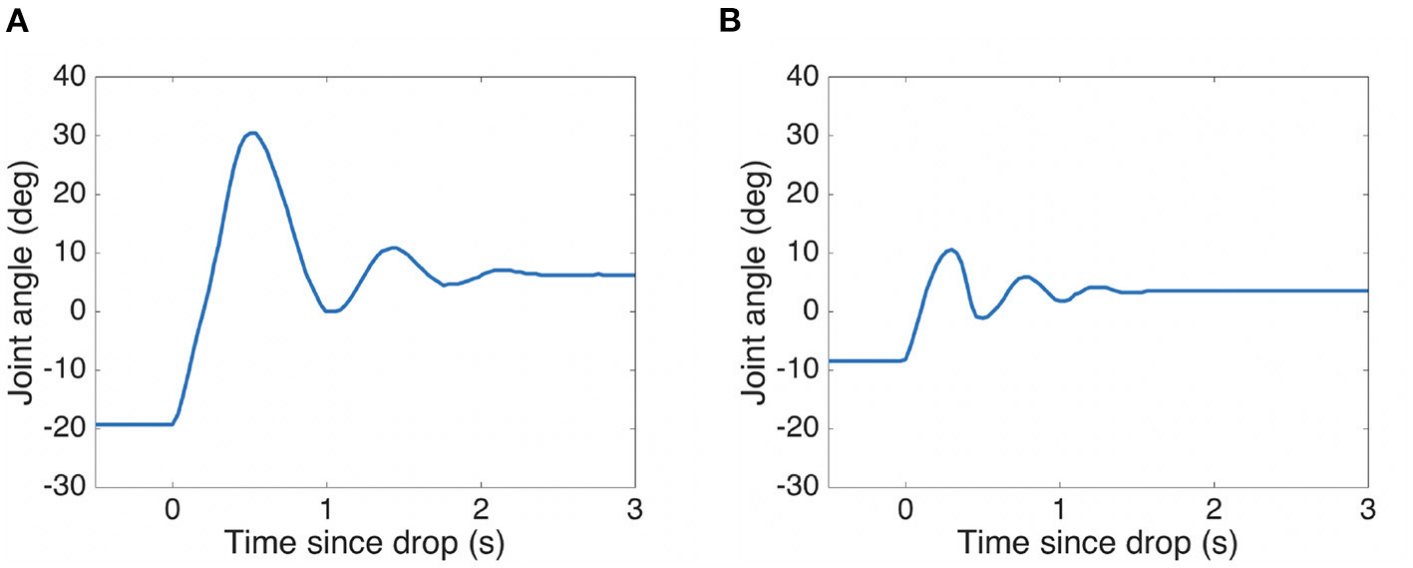
Elbow response to 1 kg mass release for two levels of co-contraction. See Figure 7 for the corresponding arm movement image series. **(A)** 25% co-contraction **(B)** 100% co-contraction.

As can be seen in Figure 8A, at 25% co-contraction the elbow joint extended to almost 20° when loaded and quickly overshot to more than 30° when the load was released, a 50° movement. This can also be seen in the full arm behavior in Figure 7A. After 2 s the oscillations had largely died out. Given that the tendons get their elasticity from polymer-based materials, instead of metallic springs, oscillations will naturally tend to die out quicker as the kinetic energy is absorbed in the polymer in the form of heat. Note that there are also other sources of potential damping here, such as friction and drag.

For comparison, the deflection with load was reduced to less than 10° with 100% co-contraction. A much smaller movement of around 20° was observed, which died out after 1.1 s, see Figures 7B,C, 8B. Thus, there is scope for using co-contraction as a way to limit unwanted large movements when a loading is rapidly released, if enough torque is available to sustain the required load at that level of co-contraction. Further work is needed to characterize the damping properties of the tendons, visco-elastic effects (such as a gradual decrease in co-contraction force with time), and the influence of co-contraction on the damping properties of the joint.

### 3.3. Exploiting Passive Compliance During Teleoperation

**Figure 9:**
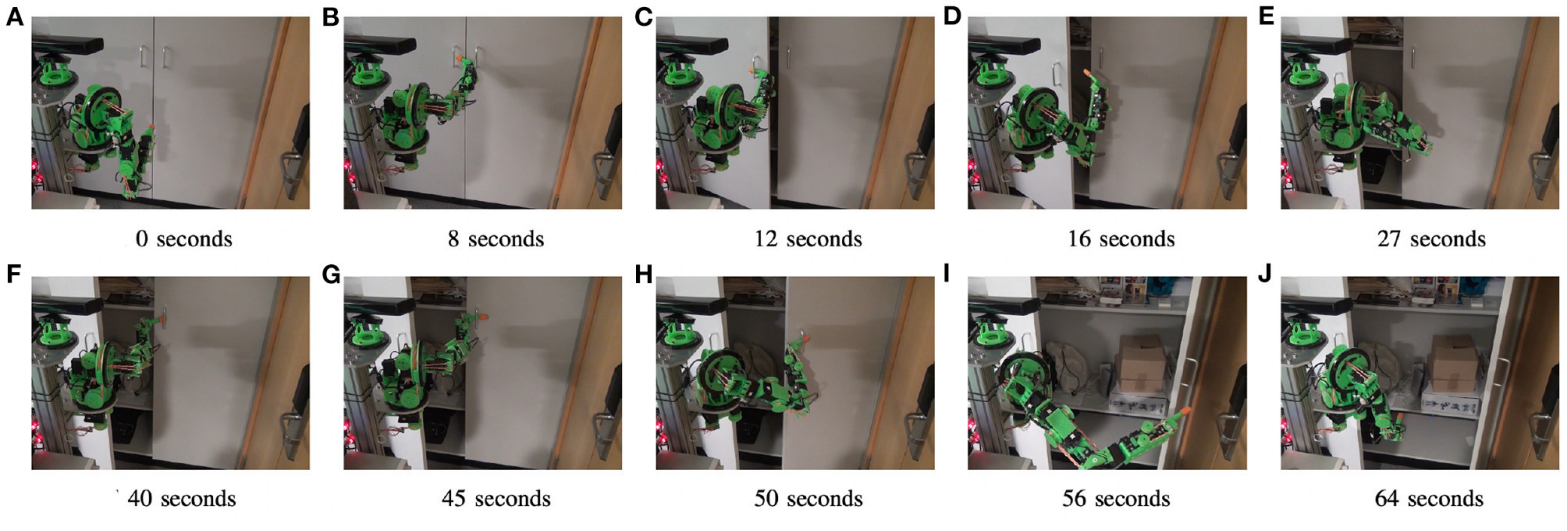
Being able to vary the level of passive compliance in real-time is useful during teleoperation with physical interactions. The GummiArm v1.0 was here teleoperated with a 3DConnexion SpaceNavigator 6 DOF input device. The task was to open both cabinet doors using only a rigid finger end-effector. The task was achieved in 64 s, helped by varying the stiffness of the arm in real-time. For example setting the arm joints loose while having the finger locked in the cabinet door handle. A high stiffness was for example used when pushing with the finger on the first door to open it fully. See full video here: https://youtu.be/QEHxqkwRZZE. **(A)** 0 s, **(B)** 8 s, **(C)** 12 s, **(D)** 16 s, **(E)** 27 s, **(F)** 40 s, **(G)** 45 s, **(H)** 50 s, **(I)** 56 s, and **(J)** 64 s.

In this test the GummiArm was teleoperated with a 3DConnexion (Boston, MA, USA) SpaceNavigator 6 DOF input device. The goal was to open cabinet doors in a typical laboratory environment with a 90 mm rigid finger as end-effector. The finger had a 30 mm patch of 3D-printed soft Filaflex plastic added for increasing friction. See Figure 9. In this experiment, we wanted to show the utility of having controllable passive compliance for such a task. The compliance is important here as the end-effector has to be jammed behind the handles of the cabinet doors for successful opening. Planning and controlling such a movement on a rigid manipulator can be very challenging due to the complex interactions between the robot arm, the finger, the handle, the doors, and the fixed cabinet itself.

As can be seen in Figure 9, the task was solved by simply pre-positioning the finger close to the handle, then maintaining a low-stiffness and open-loop control configuration while pushing the finger behind the handle, and when pulling backwards to open the doors. During the opening movement, the finger is locked between the handle and the door. The wrist thus has to be aligned correctly to follow along the pure rotation of the doors. The right level of passive compliance makes this quite easy for GummiArm. The control approach for this task was based on the basic equilibrium model in Figure 5A. The teleoperation commands from the joystick were resolved into desired joint velocities by the use of standard differential kinematics. These were then converted to desired changes to the equilibrium point *p* through a static mapping, and fed through the equilibrium model to generate commands for the actuator servos. This generated the hand velocities during the physical interaction, fine-tuned by the user using visual feedback.

The arm was then made stiff to push open the left door using the point of the rigid finger, see Figure 9C). If the arm had been kept soft, a buckling of *wrist pitch* and *elbow* joints would have been the result, complicating the completion of the task. The full video is available at https://youtu.be/QEHxqkwRZZE.

### 3.4. Performing Fast Ballistic Movements

**Figure 10:**
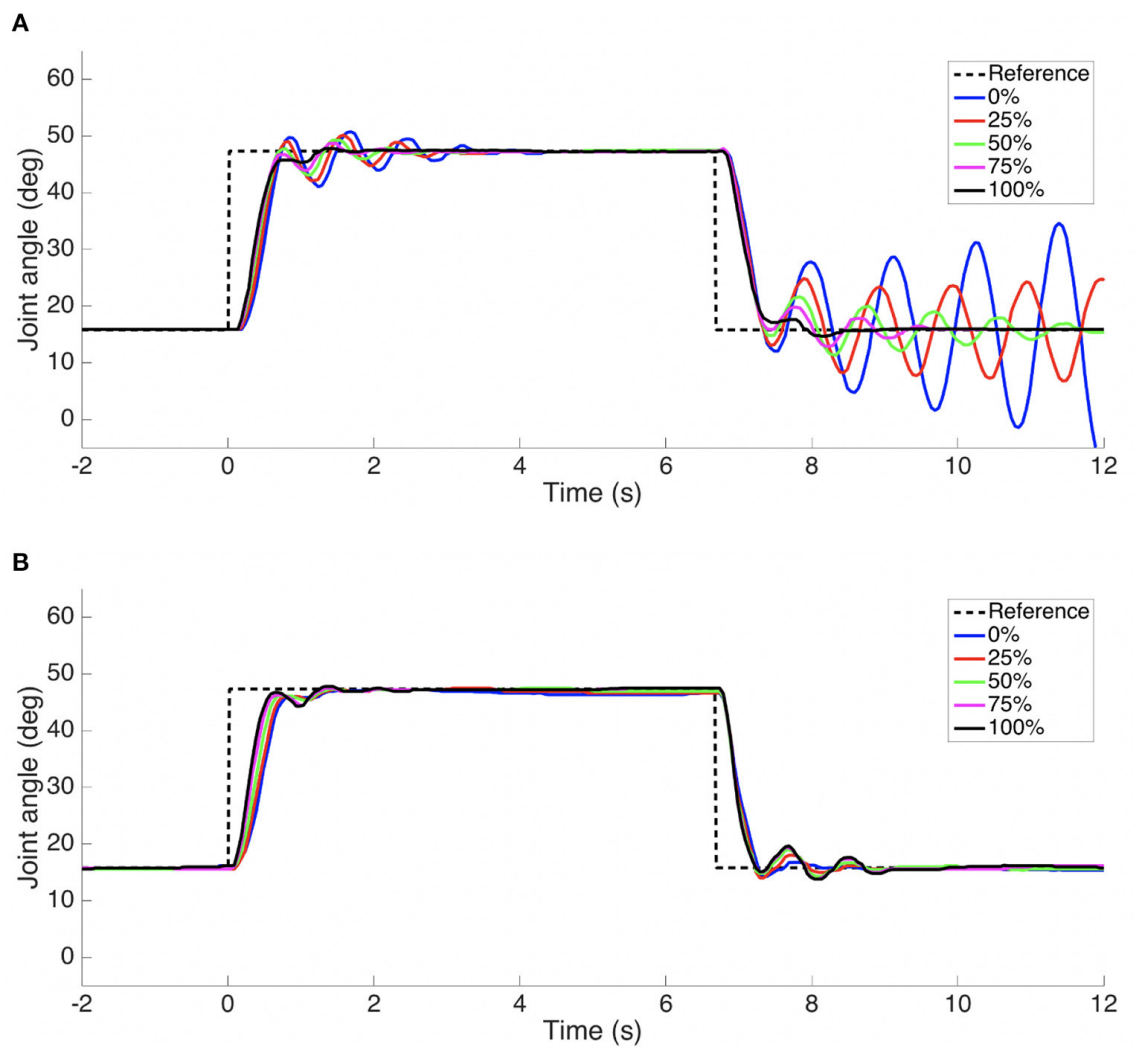
A combination of a ballistic phase and a slower feedback phase performs well even with PD gains that are not “tuned” for the situation. Step responses of the *shoulder pitch* joint with *elbow* extended and hand carrying a 275 g payload. The PD gains were tuned for the situation where the *elbow* was half-extended and the hand empty. Average of 3 attempts for each trajectory. An angle of 0 corresponds to a vertically hanging arm. **(A)** Feedback control only **(B)** Two-phase ballistic/feedback control. The percentage in the legend indicates the co-contraction level the arm had at the onset of the movement. See Figure 11A for the co-contraction profiles followed for the two-phase ballistic/feedback control.

In this experiment we compared a bio-inspired two-phase control approach with a plain feedback controller on fast step movements in joint space. A well “tuned” PD controller works well for the conditions under which it was adjusted, and if the arm maintains a high stiffness (co-contraction) throughout the movement. However, movements attempted with a low stiffness, and where the conditions have changed markedly, have a degraded performance. In this experiment the *shoulder pitch* joint was used, where a PD controller was tuned with the elbow half-extended and the hand empty. The controller was then tested with the elbow extended and hand carrying a 275 g payload. See Figure 10A for the results, averaged over 3 attempts. The performance deteriorates as the co-contraction is lowered, and instabilities are seen with 0 and 25% co-contraction.

Humans can control their arms accurately on fast point-to-point movements, and actively modify the co-contraction levels to suit the task requirements. For example by increasing co-contraction when accuracy is needed (Gribble et al., 2003). The two-phase control explored here consisted of a first open-loop ballistic phase, followed by regular PD closed-loop control when close to the target. The switch was made when a given percentage of the movement (in measured joint angle) was completed, here 50%. A simple inverse model provided a reasonable guess for the size of the initial ballistic movement to be performed, i.e., what equilibrium point *p* in Equation (1) the two opposing actuator should aim for. At the onset of a substantial movement the co-contraction level was quickly commanded to the maximum value (100%). This was regulated through a co-contraction "excitation" module, which also gradually "relaxed" the co-contraction back down to the desired level over time when no such movement had been commanded. This was done to enable the robot arm to keep operating in a soft mode for smaller movement, while exploiting the co-contraction to help in the feedback phase for larger movements.

The modifications to the control architecture can be seen in Figure 5C, and is described in more detail in Stoelen et al. (2016). As can be seen in Figure 10B, the combined ballistic/feedback controller performs well also under these conditions, adding some robustness to uncertainties. The feedback phase was needed to ensure the desired joint angle was met with a low steady-state error, as the internal model used was a simple empirical mapping from desired joint angle θ_*d*_ and co-contraction level *c* to the target equilibrium point for the ballistic phase *p*_*b*_. As can be seen in Figure 10, the oscillations are more pronounced for both controllers when returning from an elevated pose to a more vertical pose. This is likely influenced by this movement being done in the direction of the gravitational acceleration, adding kinetic energy to the system. The initial/final pose is one where the arm is close to hanging down vertically. Here the arm becomes quite challenging to control if a low co-contraction level is used, as the controller is attempting to exert influence through what is essentially two slack elastic bands.

**Figure 11:**
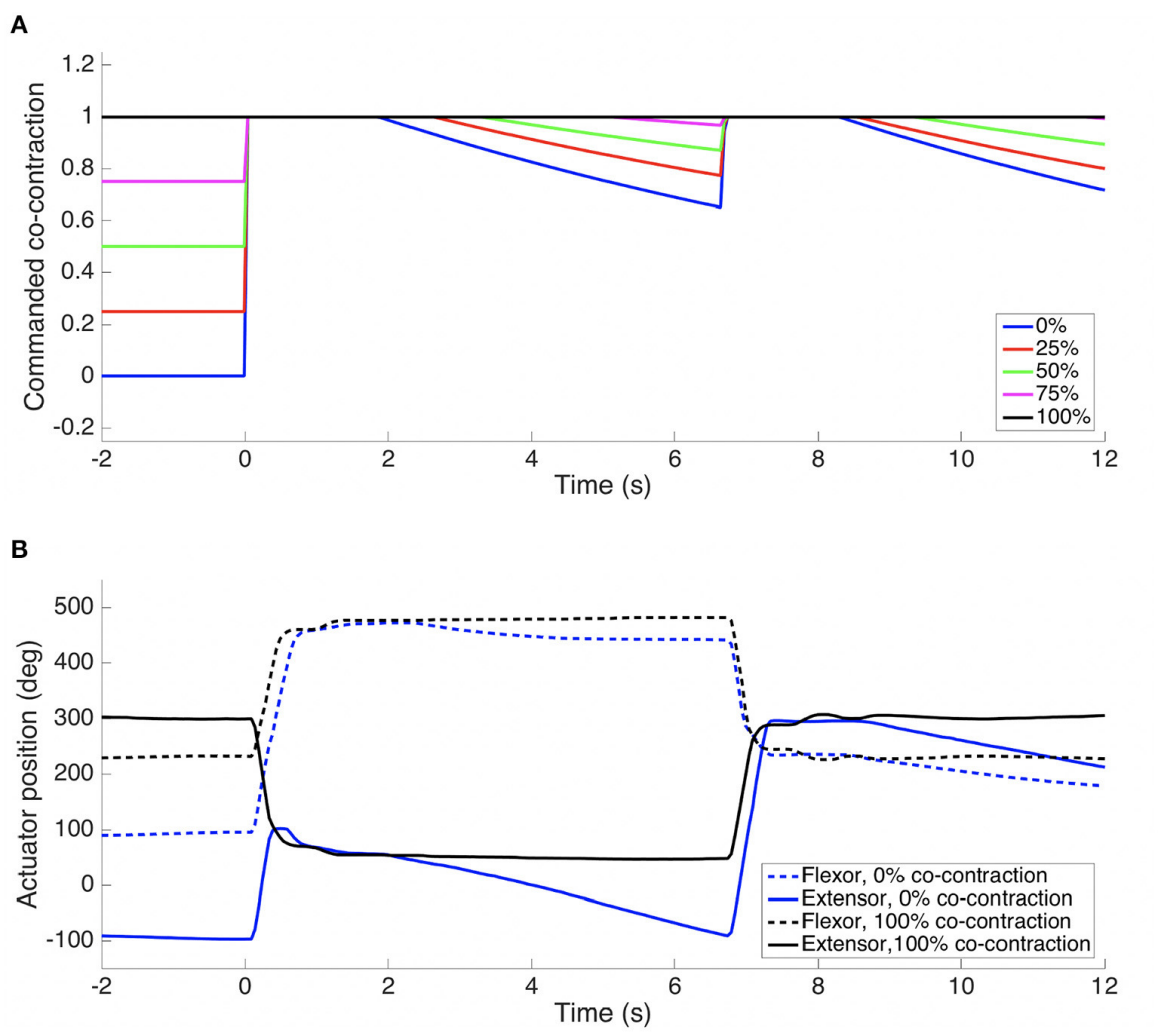
In **(A)** is seen the commanded co-contraction (c), and in **(B)** the actual actuator positions (α_*flexor*_ and α_*extensor*_), for the two-phase ballistic/feedback controller with extended arm and a 275 g mass payload. See Figure 10B for the corresponding desired and actual joint output positions (joint angles).

Figure 11A shows the corresponding developments in commanded co-contraction. The commanded co-contraction starts at different levels, but is quickly excited to the maximum of 1 (100%) for all cases. After a period, the co-contraction is slowly relaxed, while tracking the desired joint angle (see Figure 10B). For the 100% co-contraction condition, the co-contraction is maintained at this level throughout. Figure 11B shows the corresponding flexor and extensor actuator positions for 0 and 100% co-contraction. The flexor angle (dashed lines) has a similar type of behavior for both levels, but with larger movements at the 0% co-contraction level. The extensor actuator co-moves with the extensor for the 100% co-contraction level, but it opposes the movement initially for the 0% co-contraction level. That is, it quickly works to remove the slack in the tendon, which helps reduce oscillations (and the risk of instability) toward the end of each movement. It should be noted that this is only an example of how a bio-inspired control can be explored on the platform. Further work is needed to fully characterize the behavior of the system shown here.

### 3.5. Demonstrating the Robustness to Impacts

**Figure 12:**
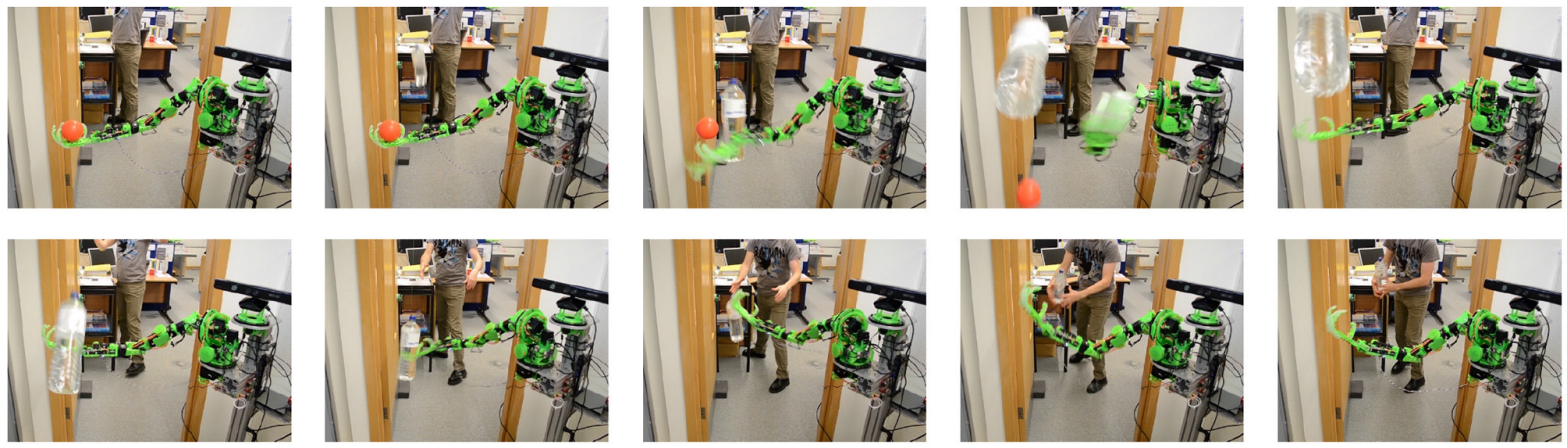
A 3D-printed robot does not necessarily mean a fragile robot. A 2 kg mass (an unopened 2 liter water bottle) attached to a string in tension was released from a height of 1.3 m above the robot arm. The mass swung down and hit the robot at the wrist when close to its point of maximum velocity. The bottle hit the arm a second time when swinging back. No damage was done to the robot, which remained fully functional after the impacts. Images taken at 0.25 second intervals, representing 2.25 seconds. See full video here: https://youtu.be/945XSTuKtAI.

According to our practical experience, load-bearing robot components made of 3D-printed PLA easily become too bulky. Although printing parameters influence the mechanical properties of parts printed using PLA, other plastics like ABS demonstrate better impact strength (Wickramasinghe et al., 2020). However, the GummiArm links are mostly interconnected by passively compliant joints and this helps reduce stress peaks and structural damage to the 3D-printed parts. Therefore, this experiment was designed to test the robustness of the GummiArm v1.0 to a high-g impact. A bottle containing 2 L of water (2 kg) suspended by a string in a pendulum configuration was dropped against the robot. See Figure 12. The distance between the point where the string was attached and the bottle's center of mass was about 1.30 m. Since the bottle was dropped from its maximum high (2.10 m) the kinetic energy available on the lowest point (0.8 m) was around 26 J.

**Figure 13:**
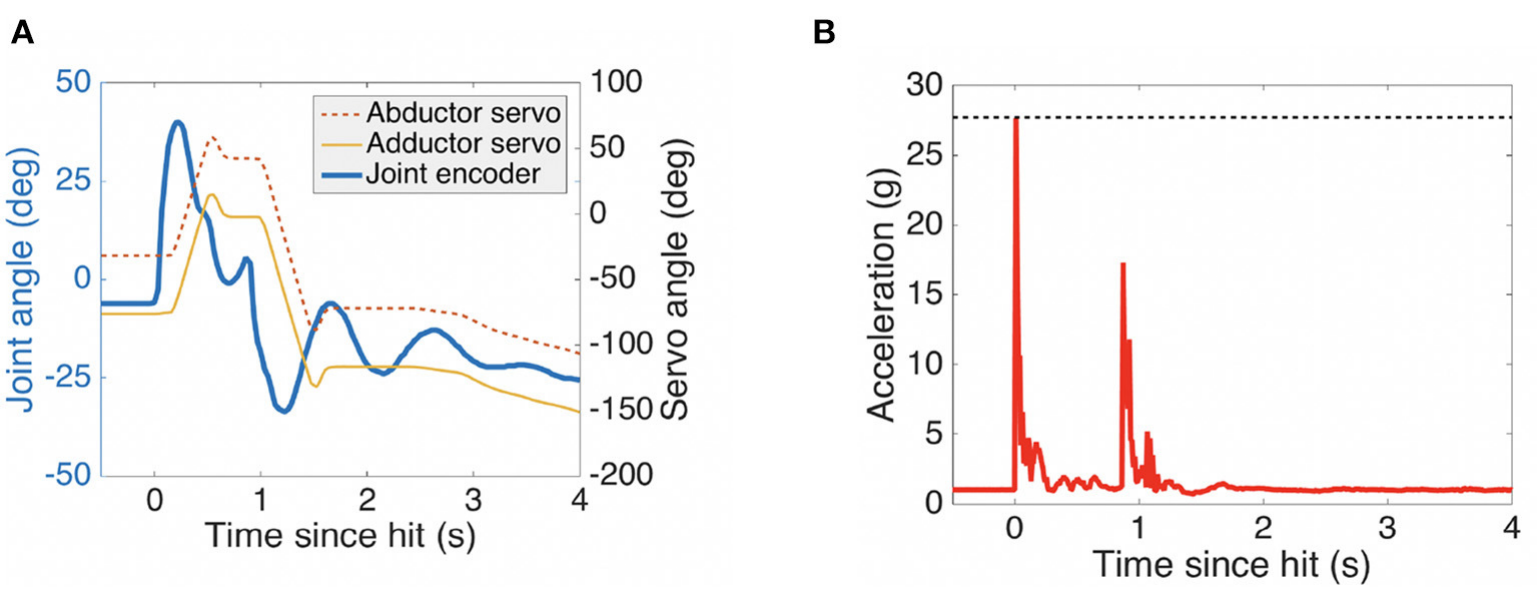
Response data corresponding to the impacts shown in Figure 12. In **(A)** the *shoulder yaw* joint can be seen to be forced through a large portion of its range of movement (“soft” range limited between –29.8 and 59.6°). The response is both passive, mainly through the elastic tendons, and active, as seen in the collision response by the servos of the joint. An accelerometer was mounted on the lower arm, and saturated at 27.8 g of acceleration during the first impact, as is seen in **(B)**. The arm was teleoperated normally from 2.5 seconds onwards.

Figure 13 shows the quantitative response of the arm. The response had a passive component through the tendon elasticity, but also an active component through a collision response behavior. This behavior consisted of shifting the equilibrium point *p* when detecting an abrupt and externally-caused joint movement. As an example, the *shoulder pitch* actuators can be seen to make a step in equilibrium point for each impact in Figure 13A. The measurements were implemented using the low-cost computer Raspberry Pi version 3 interfaced through I2C to the inertial measurement unit (IMU) Six-Axis (gyro + accelerometer) MEMS device MPU-6050 from Invensense (San Jose, CA, USA). The MPU-6050 has a user-programmable gyroscope and also a user-programmable accelerometer. The latter was setup for a full-scale range of ±16 g acceleration for each individual axis (27.8 g when considered the absolute value of all three axis) during the experiment. See Figure 13B. All the data was sent directly from the Raspberry PI using its onboard wifi interface and the WebSockets protocol together with Rosbridge at 100 Hz (code available at https://github.com/ricardodeazambuja/MPU6050_to_ROS).

### 3.6. Examples of Applications

**Figure 14:**
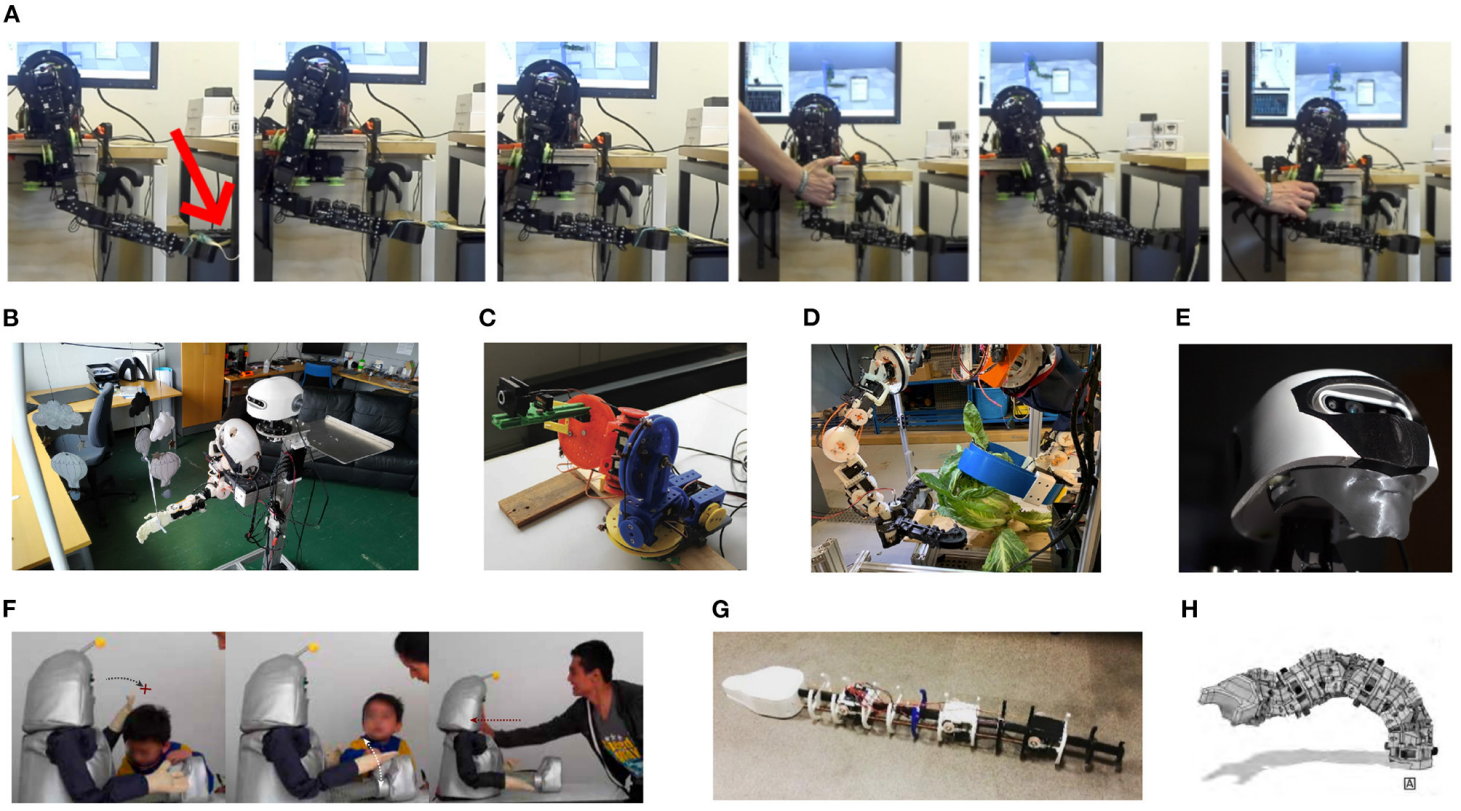
Example applications of the GummiArm, and its related concepts. **(A)** rhythmic movement generation and online frequency adaptation (Degroote et al., 2020). **(B)** exploration and exploitation of sensorimotor contingencies (Houbre et al., 2020). **(C)** visual-servoing on low-accuracy, low-cost arms (Bonsignorio and Zereik, 2020). **(D)** robust Arms for robotic cauliflower harvesting (Klein et al., 2019). **(E)** a parametric, modular and open source robot head for the GummiArm (Netzev et al., 2019). **(F)** soft and robust arms for a huggable social robot (Casas-Bocanegra et al., 2020). **(G)** a variable stiffness actuated snake (Draper et al., 2017). **(H)** a modular and stackable actuator with variable stiffness (Wilmot and Howard, 2021).

Whereas, the above experiments were performed as part of the GummiArm development at the University of Plymouth (UK), there have been a range of use of the technology in the literature. Both using the full GummiArm, only parts of the arm, or the general actuation approach. Below we outline the most relevant ones for Embodied AI, and all are visualized in Figure 14.

#### 3.6.1. Exploration and Exploitation of Sensorimotor Contingencies

In Houbre et al. (2020), the GummiArmRE was used to test a control architecture that learns sensory-motor contingencies (O'regan and Noë, 2001). The work also builds on Dynamic Field Theory (DFT), and borrows from Developmental Robotics by applying motor babbling. Actions are generated intrinsically by the system, by introducing a model to autonomously switch between exploration and exploitation behavior, based on recent progress in neuroscience regarding the role of the basal ganglia. The GummiArm was used to pull on a baby toy, with the system receiving visual input of the toy but not its own arm. For simplicity, the work limited the actions to one degree of freedom movement (the *upperarm roll*). Visual input is provided by the MaFaRo (Netzev et al., 2019) robot head. The exploitation behavior led to an increase of visual reward, while the switch between exploration and exploitation behaviors prevented the system from stagnating in one of the two. In addition, the model did not require exploring the complete action space to achieve a stable sequence of actions thanks to the switch mechanism. Further work was proposed with the whole GummiArm and a three-dimensional neural field.

#### 3.6.2. Rhythmic Movement Generation and Online Frequency Adaptation

In Degroote et al. (2020) the GummiArm was controlled using a simple two-neuron oscillator network, which acted like a Central Pattern Generator (CPG), and generated rhythmic movements for the arm. The frequency of the movement was adapted to the current arm properties and physical interaction experienced in an online fashion, using a Dual Integral Learning (DIL). The algorithm was able to achieve efficient rhythmic movements of the arm, emulating simple pick and place tasks, while adapting online to changes in arm co-contraction, load and human intervention. Thus, the system coupled the simple CPGs with the world, through the soft joints of the GummiArm, which drove the error signal used by the learning algorithm. Interestingly, Bonacchi et al. (2021) have recently shown that the elasticity of a joint can be exploited to achieve point to point goal-directed periodic movement, by tuning the elasticity to shape the natural modes of the system. The GummiArm joints could serve well for future experiments in this direction.

#### 3.6.3. Visual-Servoing on Low-Accuracy, Low-Cost Arms

In Bonsignorio and Zereik (2020) a simplified version of GummiArm, (H2Arm, see Table 1), with only 4 DOF and no position feedback on its actuators, was used to compare two different methods to control a robotic arm: Belief Space Planning (BSP) and Proportional-Integral-Derivative (PID). The aim of the work was to provide a platform and an instance, for reproducible research, enabling the assessment of the performance of control strategies in a real robotic arm with low weight, low accuracy and compliance. The work responds to a gap in the literature on real-world comparison of control methods on a robotic arm. The authors provided results for several test campaigns with more than 200 runs, under different conditions, comparing BSP and PID control. In general, the BSP method proved to be more robust and able to resolve the tasks proposed in the majority of the cases, specially when noise is introduced; while the PID method did not reach full reliability in any of the cases, and behaved poorly when noise was introduced. The work included quantitative results that can be reproduced, and was published as a "reproducible" article (IEEE Robotics and Automation Magazine), which included supplemental information, data, code and design repositories. The authors intends to evaluate deep (reinforcement) learning and changes in the platform's hardware in future work.

#### 3.6.4. Soft and Robust Arms for a Huggable Social Robot

In Casas-Bocanegra et al. (2020), a novel social robot is presented for Social Assistive Robotics (SAR). The work spans the design, development and validation of a SAR robot. The project responds to a need for SAR robots that are specifically designed for children with autism spectrum disorder, and that can safely allow physical interaction. One of the objectives of a SAR robot is to provide tactile interactions to mediate between the CwASD, its peers, and adults. The prototype developed by Casas-Bocanegra et al. (2020), CASTOR, was an open source, modular, humanoid robot, which arms are a simplification of the GummiArm. That is, using only 3 DOF, and removing the servos that provide co-contraction (and therefore the variable stiffness), which leads to Series Elastic Actuation (SEA). The design responds to the objective of creating humanoid appearance and emulating physical gestures in real applications. The case study shows that children interacted mainly with the head and the arms of the robot and the interactions included movement obstruction and strong manipulation and impact on the robot. The authors claim that the use of SEA in SAR robots has the potential for a safer and life-long interaction, as opposed to the use of stiff joints, found in the majority of current SAR robots.

#### 3.6.5. Robust Arms for Selective Cauliflower Harvesting

In Klein et al. (2019) an adaptation of the GummiArm was developed for application in cauliflower harvesting. The prototype included a wheeled platform that could be moved along planted rows, with two GummiArms hung from the top of the internal structure. To comply with the payload requirements, a bi-directional set-up of the pulley system was used in the most demanding joints. The system was composed of two arms, one for cutting and one for picking. The variable-stiffness actuation and inherent damping allowed operating close to the ground, dealing with complex plant foliage, and with a relatively simple control algorithm. The objective of the work was to respond to a need to automate harvesting of brassica produce, to reduce production costs associated to manual labor, reduce waste and increase hygiene. The proof-of-concept presented addressed most of the harvesting cycle, namely: maturity identification, curd grasping and cutting. Other research in the same group at the University of Plymouth used the technology for developing selective harvesting tomatoes and raspberries. See for example (Mohamed et al., 2019).

### 3.7. Other Usage

This section outlines other usages of the concepts and technology developed for the GummiArm. Draper et al. (2017) used the composite tendon solution from GummiArm in a variable-stiffness snake robot. The design was targeted at emulating the natural locomotion of snakes for a typical search and rescue scenario. Wilmot and Howard (2021) used similar tendons as developed for the GummiArm for a modular 2 DOF actuator with variable-stiffness that could be assembled to form a continuum-type actuator. Manchola et al. (2018) developed a low-Cost lower-leg prostheses with agonist-antagonist actuation. The system employed Dynamixel servomotors actuating composite tendons with a Filaflex core and meshed nylon around it. In Netzev et al. (2019) a parametric, modular and open source robot head for the GummiArm was developed.

Mick et al. (2019) proposed a robotic arm, (Reachy, see Table 1), suitable for research in control strategies and interfaces in human-driven robotics, such as neuroprosthesis or teleoperated robotics. The arm is fixed at the shoulder level and is designed to emulate human behavior while keeping the number of actuators low. Stemming from the Poppy project, the solution shares the same principles of a low-cost, modular, and open source design. The authors compared the arm to the GummiArm, listing their approach as more suitable for rehabilitation engineering, while the GummiArm is more suitable for bio-inspired actuation and compliant control. Vavrecka et al. (2020) developed a virtual robotic toolkit (myGym) for the propose of developing and comparing neural network architectures dedicated to reinforcement learning (RL). The toolkit serves as an extension of the popular OpenAI Gym, and it provides additions such as a simpler parameter definition, domain randomization and full visual augmentation. The toolkit includes the GummiArm, among other research robots, and is compatible with intrinsic motivation and unsupervised learning architectures.

The introduction of robotics in agriculture has the potential to increase efficiency in terms of cost and quality, while reducing the environmental impact (Duckett et al., 2018). For selective harvesting, the repetitive, dull, and precise movements required are suitable for a robot. However, challenges such as avoiding a negative impact on the crop when harvesting, dealing with noisy and incomplete sensory data in a semi-structured environment, and being safe to work along with humans, points to the desirability of physical compliance in robot arms for this application. Fieldwork Robotics Ltd (Cambridge, UK, https://fieldworkrobotics.com) was spun-out of the same research group developing the GummiArm in 2016, and has developed technology for selective harvesting of berries and other crops that draws inspiration from the GummiArm, particularly in being low-cost, modular and variable-stiffness.

## 4. Discussion

The GummiArm project has now been live for 6 years, and we have attempted to give an insight into the motivation, development, and use of the project during that period. The project has led to impact academically, by making novel experiments possible, but also economically, by inspiring variable-stiffness robots for selectively harvesting vegetables, fruits and berries. The current robot arm design hosted online is now exclusively maintained by the community created around it, and has a set of limitations that offer opportunities for future research.

The GummiArm is fairly easy to replicate, due to the largely 3D printed body. However, the low tolerances of assembly and the flexibility of the plastic pieces themselves means the accuracy of the kinematic model of the arm, which assumes rigid bodies, is degraded with external loading or poses perpendicular to gravity. Thus, any tasks demanding a high absolute accuracy of the gripper requires an approach to compensate for this, for example using visual servoing with gripper-mounted cameras (Mohamed et al., 2019). Furthermore, the Dynamixel servos provide a relatively slow and inaccurate reading of the load on the servo, which makes estimating forces and torques difficult to do in real-time. Further work on integrating force sensors, such as the ones made available from Hendrich et al. (2020), into the tendons themselves, or the pulley structure, could enable more advanced approaches for controlling the compliance during physical interaction with the environment. While the GummiArm was inspired by the rigid links of the human arm, approaches for making also the links non-rigid and variable-stiffness (see for example Mena et al., 2020) may be of interest for future iterations of the arm.

A comprehensive and long-term wear analysis has not been completed on the GummiArm. However, while operating more than 5 arms over a period of 6 years at the University of Plymouth no Dynamixel servo motors were replaced due to mechanical failures, despite the arms being used extensively for tasks involving physical interaction, such as cauliflower harvesting, selective tomato harvesting (see e.g., https://youtu.be/q8oPxP861Nw), motor babbling with physical contact (see e.g., https://youtu.be/RVkLdP-tSQA), and door opening (see e.g., https://youtu.be/ytCcpD84Jt0). As a concrete example, the same robot clone was part of two 2-day exhibits in October 2016 and June 2017, where it was setup to automatically detect bystanders, offer the hand, close the hand on detection of contact, and do a dynamic hand-shake for about 10 s. This activity involved hundreds of handshakes per day from random participants, with little or no prior instructions about the robot's physical limits to the participants. Some issues were encountered over these 4 days, but mostly limited to screws not having sufficient Screwlock to remain in place. This indicates that the composite tendon system helps protect the arm hardware from physical interaction, and shocks in particular. The composite tendons themselves have so far tended to fail at the attachment points. Some creep should also be expected for the elastomer used in the tendon core, but this has not been a significant problem for the usage seen so far.

The complete URDF files and 3D mesh files for the arm can be found in the online repository, and the arm is integrated with the MoveIt! framework (https://moveit.ros.org) for motion planning. Thus the arm can be visualized easily in RViz, and simulated in Gazebo, as a regular robot arm. At the time of developing the project there were few options available for simulating also the variable stiffness properties of the joints as part of a full robot arm, however this is currently being explored with the MuJoCo physics engine (https://mujoco.org). We believe the arm can be made suitable also for research on brain-inspired control. The length of a muscle/tendon can already be estimated from the geometry of the attachment points and the pulleys, plus the measured joint angle. This can be complemented by sensors embedded in the tendons, for example using stretch sensitive sensors based on carbon-infused elastomers (Shintake et al., 2018). These could enable bio-inspired force sensing for each tendon in a uni- or bi-directional antagonist setup. Similarly, the arm may offer interesting characteristics for replicating experiments on human motor control, such as learning activation and co-activation of muscles on tasks with both predictable and unstable components (Franklin et al., 2008). For applications where static poses with varying degrees of stiffness are important, worm-gear reduction systems can be beneficially used in conjunction with the composite tendons (Howard and Stoelen, 2021).

As seen in Table 1, the GummiArm is the open-source robot arm with the most variable-stiffness joints. While more expensive than the arms that do not offer variable-stiffness, there seems to be a great potential for further reducing the material cost of the arm. On the order of 2/3 of the current material cost of the arm is in the 6 Dynamixel MX-106T servos in the shoulder. If these could be replaced with suitable stepper motors, the overall material cost of the arm could approach $1500. This would open up new uses for the arm, including commercial and educational ones. At the same time, the payload capacity for the original GummiArm (v1.0) was quite low. It was measured to be 0.75 kg at the resting pose (300 mm forward from shoulder roll axis, see Figure 2), 0.65 kg at half forward extension (405 mm forward), and 0.45 kg at full forward extension (510 mm forward). The hand reduces this capacity further, although the main actuator for the hand is already included in the lower arm assembly. The GummiArmCE design, with a bi-directional actuator in the elbow joint has a higher payload, and above most of the arms in Table 1, but it is still below typical light industrial manipulators. Arm versions that trade a higher payload capacity for lower speed can be constructed by altering the pulley dimensions, while sacrificing speed.

We believe that promoting replication of experiments involving physical robots, and approaches for Embodied AI in particular, is needed to make real progress, and to address real-world robotics challenges. The GummiArm has inspired a set of experiments ranging from sensorimotor contingencies (Houbre et al., 2020), to using central pattern generators (Degroote et al., 2020), to huggable soft robots (Casas-Bocanegra et al., 2020), and even first attempts at truly replicable robotics experiments (Bonsignorio and Zereik, 2020). It has also inspired robots capable of picking raspberries autonomously in complex real-world scenarios. The GummiArm complements other open-source efforts in soft robotics that can help enable such replication. However, replication of existing studies needs to be seen as attractive for the individual researcher, and for the research groups. More efforts are therefore likely needed to promote this, for example in the form of special issues in the relevant outlets available for Embodied AI research.

Finally, two lessons learned the hard way: (1) managing large open source projects with both hardware and software necessitates dedicated technical staff to promote the project, maintain the website and repositories, and provide basic user support, and (2) modularity of both hardware and software should be implemented from the start, to help promote reuse of, reduce workload in administrating versions of, and increase the impact of, projects with open source robot hardware and software.

### 4.1. Conclusions and Future Work

In this paper we have presented the GummiArm Project and outlined its motivation, development and applications. The main aim of the project was to develop a platform suitable for Embodied AI research, but that also could be used in practical robotics applications where unforeseen physical interactions are possible, such as selective harvesting in horticulture and in service robotics. By being largely 3D-printable on hobby-grade printers, the arm enables a fast and iterative concurrent design loop of hardware and software, while the human-inspired variable-stiffness joints opens up interesting opportunities for Embodied AI research.

Robot hardware that is printable, open source and low-cost enables physical robot experiments to be used continuously in the design process, much like the test-driven development that is widely used in software projects. This can help quickly weed out bad design decisions and unjustified assumptions, and help make those experiments easy to replicate by other researchers. It also enables robot designers to have access to the full design space of “body” and “brain,” like in the evolution of biological agents. And like their biological counterparts, robots should also be expected to collide, slip, and miscalculate the effects of actions. The GummiArm can be made “soft” when needed, for example to respond to uncertainty in the sensory data, and the 3D-printable parts can be replaced (and improved) quickly and cheaply. We believe low-cost and open platforms are needed to objectively evaluate results in Embodied AI, by enabling the replication of experiments, and for comparing approaches qualitatively and quantitatively in terms of relative performance. That is, there is a need to comply with the scientific method to have real progress.

Six years have gone by since the inception of the GummiArm, and new research is needed to take the design further (or start something new), including force sensing in the tendons, more advanced control methods, and a stiffer mechanical structure. Further work is also needed to characterize the viscoelastic properties of the composite tendons, and to optimize the tendon design for different joints, including thickness of the core elastomer, the exact material used, and the pitch angle of the non-elastic part. We hope the open-source GummiArm genes can live on in the research community through novel arm designs, and through further applications in Embodied AI research.

## Data Availability Statement

The original contributions presented in the study are included in the article/supplementary materials, further inquiries can be directed to the corresponding author/s.

## Author Contributions

MS and BL: conceptualization and writing–original draft. RA, FB, and AC: conceptualization and writing–review. All authors contributed to the article and approved the submitted version.

## Funding

The GummiArm was initiated by a Marie Curie Intra-European Fellowship within the 7th European Community Framework Programme (DeCoRo FP7-PEOPLE-2013-IEF) at the Centre for Robotics and Neural Systems (CRNS), University of Plymouth, UK. The project also had synergies with Automated Brassica harvesting in Cornwall, a sub-project of Agritech Cornwall, part-funded by the European Regional Development Fund (ERDF), and with match-funding from Cornwall Council, UK. Writing of the manuscript and production costs have been funded through the project Teknoløft Sogn og Fjordane from the Norwegian Research Council under grant no. 22071.

## Conflict of Interest

FB is employed by Heron Robots (Genova, Italy). MS is also Director, Chief Science Officer (CSO) and shareholder in Fieldwork Robotics Ltd. (Cambridge, UK), which was spun-out of the original research group at the University of Plymouth to develop robotic solutions for selective harvesting. The work has led to a pending patent on improvements to the hardware and control of passively compliant robotic arms for applications such as agriculture (MS-US Patent App. 16/646,565, 2020). The GummiArmRE robots are no longer offered commercially by any of the authors. The remaining authors declare that the research was conducted in the absence of any commercial or financial relationships that could be construed as a potential conflict of interest.

## Publisher's Note

All claims expressed in this article are solely those of the authors and do not necessarily represent those of their affiliated organizations, or those of the publisher, the editors and the reviewers. Any product that may be evaluated in this article, or claim that may be made by its manufacturer, is not guaranteed or endorsed by the publisher.
